# PROTOCOL: Multiagency programmes with police as a partner for reducing radicalisation to violence

**DOI:** 10.1002/cl2.1110

**Published:** 2020-08-05

**Authors:** Lorraine Mazerolle, Adrian Cherney, Elizabeth Eggins, Angela Higginson, Lorelei Hine, Emma Belton

**Affiliations:** ^1^ School of Social Science University of Queensland, St. Lucia Campus St. Lucia Queensland Australia; ^2^ School of Justice Queensland University of Technology, Gardens Point Campus Brisbane Queensland Australia

## 1 BACKGROUND

### 1.1 The problem, condition or issue

Violent radicalisation is a complex problem, complicated by the lack of a clear terrorist profile and variation in the risk factors that predict violent extremism across individuals and groups (Campelo, Oppetit, Neau, Cohen, & Bronsard, 2018; Carlsson et al., 2020; Desmarais, Simons‐Rudolph, Brugh, Schilling, & Hoggan, 2017; Wolfowicz, Litmanovitz, Weisburd, & Hasisi, 2019). While models of understanding radicalisation vary (Borum, 2015; Christmann, 2012; Desmarais et al., 2017; Horgan, 2008; Koehler, 2017; Kruglanski, Bélanger, & Gunaratna, 2019; Sarma, 2017), it is broadly defined as the process by which a person adopts extremist views and moves towards committing a violent act (Irwin, 2015; Jensen, Atwell Seate, & James, 2018). Radicalisation has been linked with individual and group engagement in terrorist attacks against innocent civilians (Wilner & Dubouloz, 2010), as well as individuals entering conflict zones to join formal extremist groups to engage in violent combat (Lindekilde, Bertelsen, & Stohl, 2016). As a result, radicalisation has become a key focus for counterterrorism and violence prevention interventions.

The complex and varied nature of individuals' progression from radicalisation to violence presents challenges for designing and evaluating appropriate interventions and policy responses (Hafez & Mullins, 2015; Helmus et al., 2017; Horgan & Braddock, 2010; Horgan, 2008; Jensen et al., 2018; Kruglanski et al., 2019). This level of complexity has driven national counterterrorism policy agendas to adopt intersectoral and multiagency responses that aim to address various radicalisation processes and risks (Beutel & Weinberger, 2016). These multiagency responses often involve partnerships and collaborations between various different agencies and entities (Hardy, 2018), such as governmental agencies, private businesses, community organisations and service providers.

Multiagency interventions can provide a framework for pooling and sharing resources to address a common problem (Crawford, 1999; Rosenbaum, 2002), such as radicalisation to violence. Yet they can be challenging to implement, and their effectiveness may be influenced by the quality and nature of the collaboration between agencies (see Berry, Briggs, Erol, & van Staden, 2011, for review; Atkinson, 2019; Gittell, 2006; Kelman, Hong, & Turbitt, 2013; McCarthy & O'Neill, 2014; Rosenbaum 2002). Multiagency interventions may be conceptualised on a continuum, with activities ranging from minimal collaboration to a wholistic integration of agencies and organisations (Atkinson, 2019). As a result, the outcomes of multiagency interventions may vary depending on the where the intervention falls—or is perceived to fall—on this collaborative continuum (Atkinson, 2019). Partnerships can enhance formal and informal communication, trust, respect, shared goals and knowledge (Bond & Gittell, 2010). Conversely, partnership‐based interventions may highlight a number of shortcomings in service delivery including the disjointed nature of services, the need for significant stakeholder buy‐in, the isolation for some of the organisations or individuals, and the resource‐intensive nature of many of these collaborations (Atkinson, 2019; Bond & Gittell, 2010; Crawford, 1999; McCarthy & O'Neill, 2014; Yousanamouth, 2019). There is also the possibility that multiagency approaches could lead to adverse outcomes (Galloway, 2017; Norton, 2018). For example, multiagency responses that have poor levels of coordination and communication could lead to cases falling through the cracks where no one agency responds under the misguided assumption that another partner agency is taking the lead (Richards, 2017; Smith, Laszlo, Ayers, & Smith, 1992). Ransley (2016) also raises the possibility of increased coercion from multiagency responses. Therefore, when assessing the effectiveness and the intended outcomes of multiagency interventions, it is also important to consider the context, potential backfire effects and quality of the processes underpinning multiagency collaboration.

A broad range of agencies and experts can be involved in multiagency approaches for reducing radicalisation to violence or violent extremism (Crawford, 1999). Nevertheless, the public police are often one of the first points of contact with individuals who have radicalised to extremism. The public police are also the first point of call for those who are concerned about or report known associates, friends or family members as being at risk of radicalisation. As such, police are important partners for identifying, reducing and building resilience to radicalisation (Cherney, 2015). This review will, therefore, focus on the effectiveness of police‐involved multiagency interventions for reducing radicalisation to violence and improving multiagency collaboration.

### 1.2 The intervention

Multiagency interventions are generally characterised by two or more entities partnering to solve a shared problem. These entities may be government agencies (such as education, immigration, customs, home affairs, employment, housing, health), or nongovernmental agencies, including: local councils, businesses, community organisations (such as churches, mosques and other houses of worship) and service providers (such as resettlement agencies, local health providers). This review will include any multiagency intervention, where at least one of those partners is the public police and where the intervention explicitly aims to address terrorism, violent extremism, or radicalisation to violence. This type of intervention can include a range of approaches, including: police engaging with different community and agency stakeholders to help identify terrorist threats (Innes, Roberts, & Innes, 2011; Ramiriz, Quinlan, Malloy, & Shutt, 2013); police working with other agencies to refer, assess, or case‐manage individuals convicted of terrorism or identified as at‐risk for radicalisation (Cherney & Belton, 2019); or police forming task forces or partaking in regular structured meetings with other agencies to problem‐solve issues pertaining to radicalisation or extremism (Koehler, 2016).

### 1.3 How the intervention might work

Some observe that there is a great deal of heterogeneity in the risk factors and triggers for radicalisation (Dalgaard‐Nielsen, 2010, 2018; Horgan 2009). This means there is a variety of risk and background factors that may lead an individual (or a group of individuals) to radicalise to violent extremism (Campelo et al., 2018; Carlsson et al., 2020; Vergani, Iqbal, Ilbahar, & Barton, 2018). Research also demonstrates the complex nature of different progression pathways from radicalisation to violence (Horgan, 2008; Kruglanski et al., 2019; Kruglanski, Webber, & Koehler, 2020). The literature is, therefore, in agreement that the complex nature of radicalisation risk and pathway processes to violence makes it impossible for any single agency, organisation or entity to address the problem alone (Dalgaard‐Nielsen, 2018). As such, interventions to address the problem of radicalisation to violence are often characterised by multiagency partnerships, working across different service delivery sectors (see e.g., Cherney & Belton, 2019; Crawford, 1999; Innes et al., 2011).

Multiagency partnerships may disrupt pathways from radicalisation to violence by collectively addressing multiple risk factors in a holistic and coordinated manner (Butt & Tuck, 2014). The multiagency approach to tackling violent extremism may be effective because it fosters a coordination of effort (Crawford, 1999; Kelman et al., 2013), draws from a broad range of expertise (Crawford, 1999), allows for information and intelligence sharing (Cherney, 2018; Murphy, 2008; Slayton, 2000) and enables the pooling of resources (Crawford, 1999; Sestoft, Hansen, & Christensen, 2017).

El‐Said (2015) describes a range of different ways that multiagency partnerships are created: by formal and informal arrangements, such as legislative or regulatory frameworks, memoranda of understanding or policy standards stipulating channels for information sharing or better interpersonal relations between agencies (see also Koehler, 2016). These arrangements create opportunities for referrals being made from various sources (Koehler, 2017), increasing the capacities for partnerships to detect and respond to those at early pathways to radicalisation and violence. The capacities of multiagency partnerships to better detect and respond to problems over and above what is possible by agencies or entities working alone are enhanced through better information sharing and referral processes (Cherney, 2018; Murphy, 2008; Slayton, 2000). Partnerships can enhance programme planning and design so that counter radicalisation strategies address the required risks and vulnerabilities among individuals and groups (Koehler, 2017). The range of expertise across multiagency partners also help to enhance programme implementation by ensuring the all required components of a strategy are delivered (Crawford, 1999). They are likewise important in relation to programme evaluation by enabling the sharing of data that can be used to assess programme effectiveness (Cherney, 2018).

### 1.4 Why it is important to do the review

Police cannot tackle the problem of radicalisation, violent extremism and terrorism on their own (Cherney & Hartley, 2017). Many of the risk factors for radicalisation and violent extremism are complex (Dawson, Amarasingam, & Bain, 2016; Hafez & Mullins, 2015; Kruglanski et al., 2019, 2020). Research suggests that is not just the presence of risk factors, but rather the accumulation of risk factors (Campelo et al, 2018; Carlsson et al., 2020; Simi, Sporer, & Bubolz, 2016) and what Vergani et al. (2018) describe as the push, pull and personal nature of the radicalisation process. The complexity of the process, therefore, can trigger a range of different vulnerabilities. Some of these vulnerabilities relate to a lack of sense of belonging (Harris‐Hogan, 2014), which requires different institutional responses spanning the family, educational and work context, all of which contribute to the formation of a sense of identity (Kruglanski et al., 2019).

The complexity and variability of the radicalisation process provides an opportunity for police to partner with various agencies and community groups to tackle radicalisation in a multifaceted manner. As such, multiagency interventions have become an important approach to tackle the problem of radicalisation and violent extremism (Butt & Tuck, 2014; Mucha, 2017; Sestoft et al., 2017). Existing evidence, however, does not provide a clear understanding of the effectiveness of police‐involved, multiagency approaches to radicalisation (Cherney & Hartley, 2017; Koehler, 2017; MacDonald, 2002). In addition, there are no existing reviews of multiagency programmes, with police as partner, for addressing radicalisation to violence.^1^ Given the cost of forming multiagency interventions and the organisational complexities of managing and maintaining these types of responses, it is imperative to know whether current multiagency approaches that include police partners are effective for reducing radicalisation to violence and enhancing multiagency collaboration. Policy‐makers, practitioners and researchers also need to understand not only whether the intervention works, but also how the intervention works (mechanisms), under what conditions or contexts (moderators), and what the implementation considerations and cost implications are.

This review will fill a significant gap in the evidence‐based literature for countering violent extremism in two ways. First, by quantitatively synthesising the existing evidence for the impact of multiagency police‐involved programmes on reducing violent radicalisation or enhancing multiagency collaboration. Second, by qualitatively synthesising research that reports on the mechanisms, moderators, implementation considerations and economic information pertaining to police‐involved multiagency programmes to counter radicalisation to violence. The results from this review will inform future decision‐making regarding the design and evaluation of multiagency programmes by synthesising the evidence for their effectiveness, identifying potential gaps in the evidence‐base and providing insight into what level of investment is required for the implementation and evaluation of primary studies.

## 2 OBJECTIVES

The first objective of this review (Objective 1) is to answer the question: how effective are multiagency interventions with police as a partner at reducing radicalisation to violence or improving multiagency collaboration? If there is sufficient data, the review will also examine whether the effectiveness of these interventions varies by the following factors: geographical location, target population, nature of the intervention approach (e.g., number of components, specific intervention techniques), and number and type of multiagency partners. The second objective of this review (Objective 2) is to qualitatively synthesise pertinent information about *how* the intervention might work (mechanisms), under what *context* or conditions the intervention operates (moderators), the implementation issues and economic considerations.

## 3 METHODOLOGY

### 3.1 Criteria for including and excluding studies

#### 3.1.1 Types of study designs

To fulfil the objectives of this review, two types of studies will be included. The specific type of studies used to address each review objective may overlap, and are detailed in the subsections below.

#### 3.1.2 Types of study designs for review of effectiveness (Objective 1)

To be included in the review of effectiveness (Objective 1), a study must be a quantitative impact evaluation that employs a randomised experimental (e.g., randomised controlled trials) or a quasiexperimental design with a comparison group that does not receive the intervention. Eligible comparison groups are: “business‐as‐usual” treatment, no intervention, or an alternative intervention (treatment‐treatment designs).

Rigorous quasiexperimental studies can also be used to estimate causality, particularly when the research design includes strategies to minimise threats to internal validity (see Farrington, 2003; Shadish, Cook, & Campbell, 2002). Strategies for reducing threats to internal validity may include: controlling case assignment to treatment and comparison groups (regression discontinuity), matching characteristics of the treatment and comparison groups (matched control), statistically accounting for differences between the treatment and comparison groups (designs using multiple regression analysis), or providing a difference‐in‐difference analysis (parallel cohorts with pre‐ and posttest measures). The following “strong” quasiexperimental designs will be eligible for this review:

- Cross‐over designs
- Regression discontinuity designs
- Designs using multivariate controls (e.g., multiple regression)
- Matched control group designs with or without preintervention baseline measures (propensity or statistically matched)
- Unmatched control group designs without preintervention measures where the control group has face validity
- Unmatched control group designs with prepost intervention measures which allow for difference‐in‐difference analysis
- Short interrupted time‐series designs with control group (<25 pre‐ and 25 postintervention observations (Glass, 1997)
- Long interrupted time‐series designs with or without a control group (≥25 pre‐ and postintervention observations (Glass, 1997)
  Less rigorous quasiexperimental designs can be used to illustrate the magnitude of the relationship between an intervention and an outcome, yet have limitations for establishing causality. Therefore, we will exclude the following weaker quasiexperimental designs in the synthesis of intervention effectiveness:
- Raw unadjusted correlational designs where the variation in the level of the intervention is compared to the variation in the level of the outcome; and
- Single group designs with pre‐ and postintervention measures.

#### 3.1.3 Types of study designs for review of mechanisms, moderators, implementation and economic considerations (Objective 2)

To be included in the qualitative synthesis of the potential mechanisms, moderators, implementation issues and economic considerations related to the intervention (Objective 2), each study must either be (a) already included in the quantitative synthesis impact evaluations (see above for review Objective 1); or (b) be an empirical study reporting on an eligible intervention. To be an empirical study, the authors must either be reporting on primary quantitative or qualitative data or conducting secondary analysis of primary quantitative or qualitative data. We acknowledge that qualitative studies may not be present “data” per se, but report on empirical work such as textual themes from key‐informant interviews or focus groups, or information gathered by observational methods (e.g., participant‐observers). Purely theoretical work, opinion pieces or research reports that only summarise, reference or describe previous intervention studies will not be used for the qualitative synthesis. However, this type of document may be harvested for relevant references.

#### 3.1.4 Types of participants

For both the review of effectiveness (Objective 1) and the review of mechanisms, moderators, implementation and economic considerations (Objective 2), this review will include studies that use any of the following populations:

1. Individuals of any age, gender or ethnicity
2. Micro places (e.g., street corners, buildings, police beats, street segments)
3. Macro places (e.g., neighbourhoods, communities, police districts)

#### 3.1.5 Types of interventions

For both the review of effectiveness (Objective 1) and the review of mechanisms, moderators, implementation, and economic considerations (Objective 2), this review will include any police‐involved multiagency intervention that aims to address terrorism, violent extremism or radicalisation to violence. Specifically, each study must meet two intervention criteria:

1. Report on a multiagency intervention where police are a partner, defined as some kind of a strategy, technique, approach, activity, campaign, training, programme, directive or funding/organisational change that involves police and at least one other agency (Higginson, Eggins, Mazerolle, & Stanko, 2015). Police involvement is broadly defined as:
  - Police initiation, development or leadership;
  - Police are recipients of the intervention or the intervention is related, focused or targeted to police practices; or
  - Delivery or implementation of the intervention by police.
  The other agencies or entities involved in the intervention may be government or nongovernmental agencies, including government agencies (such as education, immigration, customs, home affairs, employment, housing, health), local councils, businesses, communities (such as churches, mosques and other houses of worship), and services providers (such as resettlement agencies, local health providers).
**AND**
2. Report on a multiagency intervention with police as a partner that *aims to address terrorism*, *violent extremism*, or *radicalisation to violence*, as defined or specified by study authors.

We anticipate that multiagency interventions with police as a partner that aim to address terrorism, violent extremism or radicalisation to violence may include:

- Police being trained OR police training or educating partner(s), to improve recognition, referral and responses to radicalisation, including guiding at risk populations towards numerous forms of support services offered by various partnerships, such as life skills mentoring, anger management sessions and cognitive/behavioural therapy (Home Office, 2015a).
- Community awareness programmes or training delivered to police OR police delivering community awareness training or programmes to partner(s) to help partner(s) identify someone who may already be engaged in illegal terrorist‐related activity and are referred to the police (Home Office, 2009).
- Police working in partnership with universities to train, engage, intervene and consult on action plans to reduce at‐risk youth to extremist messaging (Angus, 2016).
- Approaches that involve police working with other agencies to refer, assess, or case‐manage individuals convicted of terrorism or identified as at risk of radicalisation (Cherney & Belton, 2019).
- Police partnering with other agencies to address radicalisation or extremism through regular structured/unstructured focus groups or meetings that may or may not be formalised (e.g., memoranda of understanding) or by forming task forces or multiagency intervention teams.
- Police working with external agencies to divert an individual away from violent extremism (e.g., UK Channel programme; Home Office, 2015b).
- Police officers undertaking various forms of engagement with different community and agency stakeholders to help identify terrorist threats (Innes et al., 2011; Ramiriz et al., 2013).

#### 3.1.6 Types of outcome measures

##### Types of outcome measures for review of effectiveness (Objective 1)

For the review of effectiveness (Objective 1), we will include studies with two main categories of outcomes. The first is radicalisation to violence. For the purposes of this review, radicalisation to violence is defined as the process by which a person adopts extremist views and moves towards committing a violent act (Hardy, 2018; Jensen et al., 2018). It is important to note that “radicalisation” remains inconclusively defined in the literature (Heath‐Kelly, 2013) and violence is just one potential outcome of radicalisation (Angus, 2016; Hafez & Mullins, 2015; Schmid, 2013). We also recognise that terminology (e.g., radicalisation and extremism) in the extant literature is often used interchangeably (Borum, 2012), and that outcomes may not be labelled explicitly as “radicalisation to violence”. Other labels that may be used include: radicalisation (Horgan, 2009), extremism, violent extremism (Khalil & Zeuthen, 2016), political violence, ideologically motivated violence, political extremism (Lafree, Jensen, James, & Safer‐Lichtenstein, 2018), violent radicalisation (Bartlet & Miller, 2012) and terrorism (Christmann, 2012).

We will include outcome data that are measured through self‐report instruments, interviews, observations and/or official data (e.g., contact with police, calls‐for‐service reporting incidents, arrests, charges, prosecution, sentencing and correctional data). Some examples of how radicalisation to violence can be measured include:

- Violent Extremist Risk Assessment‐2: a risk assessment of the likelihood of violence by an offender who has been convicted of ideologically motivated violence (Pressman & Flockton, 2012).
- Extremist Risk Guidance Factors (ERG 22+): assesses the needs and risks of offenders who have either been convicted of an extremist offence or have shown behaviours or attitudes that raise concerns about their potential to commit extremist offences (Knudsen, 2020).
- IAT‐8: Assesses the effectiveness of a current intervention at reducing or altering the level of vulnerability to radicalisation (RTI International, 2018).
- RADAR assessments: identifies individuals who would benefit from services to help them disengage from violent extremism by assessing a variety of observations including religious understanding and knowledge, radicalisation source, intervention goals and progress undertaken to achieve these goals (Cherney & Belton, 2019)
- Terrorist Radicalisation Assessment Protocol (TRAP‐18): a professional judgement instrument for risk and threat assessment of individuals who may engage in lone‐actor terrorism (Meloy, 2018).

The second category of outcomes that will be included in the review of effectiveness is multiagency collaboration, broadly defined as a measure that relates to the quality and nature of the partnership between the agencies involved in the intervention. The quality and nature of collaborations or partnerships can be operationally defined in different ways ranging from the degree of practical sharing of resources (Rosenbaum, 2002) to relational perspectives that encompass variables such as: frequency and quality of communication, shared goals and knowledge, and trust or respect (Bond & Gittell, 2010; Gittell, 2006). This review will include both practical and relational measures of collaboration, which may be captured by self‐report or official/administrative data in one or more of the following categories:

- Information sharing (e.g., frequency, quality);
- Perceptions of trust, respect, or legitimacy within multiagency collaborations; or
- Degree of shared goals and understanding between multiagency partners.

##### Types of outcome measures for review of mechanisms, moderators, implementation and economic considerations (Objective 2)

To be included in the qualitative synthesis of the potential mechanisms, moderators, implementation issues and economic considerations (Objective 2), no specific outcome measures are required. Specifically, any empirical study of a police‐involved multiagency programme that aims to address terrorism, violent extremism or radicalisation to violence will be examined for qualitative or quantitative data pertaining to potential mechanisms, moderators, implementation or economic considerations (see Supporting Information Appendix C for definitions). We note the differences in the conceptualisation of “outcomes” for quantitative and qualitative studies, whereby qualitative studies may not distinguish between different types of variables such as independent, predictor, outcome, moderator, or mediator variables. Rather, qualitative studies are likely to present thematic textual data drawn from interviews, focus groups or observational methods. In addition, study authors may use mechanism, moderator, implementation, and economic variables as outcome variables or they may use data within these domains as mediators or moderators to explore their impact on study outcomes. To provide a comprehensive synthesis of potential mechanisms, moderators, implementation issues, and economic considerations, we will include empirical studies that report on data in any of these domains, regardless of whether the data is conceptualised as an “outcome variable”.

##### Duration of follow‐up

For both the review of effectiveness (Objective 1) and the review of mechanisms, moderators, implementation and economic considerations (Objective 2), we will include studies with follow‐up periods of any length. If there is variation in the length of follow‐up across studies, we will group and synthesise studies with comparable follow‐up durations. For example, short (e.g., 0–3 months postintervention), medium (>3, <6 months), and long‐term follow‐up (>6 months postintervention).

##### Types of settings

For both the review of effectiveness (Objective 1) and the review of mechanisms, moderators, implementation and economic considerations (Objective 2), we will include studies that report on an impact evaluation of an eligible intervention using eligible participants, outcome(s) and an eligible research design in any setting. All geographical regions will be included in the review (i.e., high‐, middle‐ and low‐income countries). If we identify a range of conceptually distinct settings, we will synthesise the studies within the settings separately.

We will include studies written in any language. At the title and abstract screening stage, we will use Google Translate to identify whether a non‐English language study is potentially eligible for review. If eligible after title and abstract screening, we will again use Google Translate using multiple sections of the document to determine its eligibility for the review. If eligibility cannot be unequivocally determined with this approach, we will call upon our international network of colleagues for assistance with full‐text screening and coding if required. We will include studies disseminated between 2002 and 2018 in the review.

### 3.2 Search strategy

We will use a common search strategy for the review of effectiveness (Objective 1) and the review of mechanisms, moderators, implementation and economic considerations (Objective 2). The search for this review will be led by the Global Policing Database (GPD) research team at the University of Queensland (Elizabeth Eggins and Lorraine Mazerolle) and Queensland University of Technology (Angela Higginson). The University of Queensland is home to the GPD (see http://www.gpd.uq.edu.au), which will serve as the main search location for this review. The GPD is a web‐based and searchable database designed to capture all published and unpublished experimental and quasiexperimental evaluations of policing interventions conducted since 1950. There are no restrictions on the type of policing technique, type of outcome measure or language of the research (Higginson et al., 2015). The GPD is compiled using systematic search and screening techniques, which are reported in Higginson et al. (2015) and summarised in Supporting Information Appendices A and B. Broadly, the GPD search protocol includes an extensive range of search locations to ensure that both published and unpublished research is captured across criminology and allied disciplines.

The GPD systematic search uses a broad range of policing and research search terms and systematically progresses the screening of the captured research in sequential stages with increasing specificity. At the initial title and abstract screening stage, records identified by the systematic search are screened on whether they are broadly about police or policing (see Higginson et al., 2015). At subsequent full‐text screening stages, documents for records screened as being potentially about police or policing are then screened on whether they report on a quantitative impact evaluation of an intervention relating to police or policing, with no limits on outcome measures. As a result, refined corpuses of policing research can be searched and extracted from the GPD without the need to use policing search terms. Because our review will capture both quantitative and qualitative studies of eligible interventions, we will extract data from the GPD from the point of title and abstract eligibility (i.e., is the document broadly about police or policing). We will search the title and abstracts within this corpus between 2002 and 2018 using the following search terms: *terror* OR extrem* OR *radical*.

Informed by the Mazerolle et al. (2020) systematic review, we will also employ additional strategies to extend the GPD search. These include:

- Searching trial registries (as listed by WHO https://www.who.int/ictrp/network/primary/en/) and Office for Human Research Protections (https://www.hhs.gov/ohrp/international/clinical-trial-registries/index.html);
- Searching counterterrorism organisation websites (see Table 1);
- Conducting reference harvesting on both the corpus of eligible documents and existing reviews;
- Forward citation searching for all eligible documents;
- Liaising with the Five Country Research and Development Network (5RD), and the Department of Homeland Security Advisory Board network for the Campbell Collaboration grants, to enquire about eligible studies that may not be publicly available;
- Personally contacting prominent scholars in the field and authors of eligible studies to enquire about eligible studies not yet disseminated or published; and
- Hand‐searching the 12‐months prior to December 2018 for the following journals to identify eligible documents yet to be indexed in academic databases:
  a. Critical Studies on Terrorism
  b. Dynamics of Asymmetric Conflict
  c. Intelligence and Counter Terrorism
  d. International Journal of Conflict and Violence
  e. Journal for Deradicalization
  f. Journal of Policing
  g. Perspectives on Terrorism
  h. Police Quarterly
  i. Policing—An International Journal of Police Strategies and Management,
  j. Policing and Society
  k. Sciences of Terrorism and Political Aggression
  l. Studies in Conflict and Terrorism
  m. Terrorism and Political Violence

**Table 1:** Grey literature search locations

Organisation	Website
Global Terrorism Research Centre (Monash University)	http://artsonline.monash.edu.au/gtrec/publications/
Triangle Centre on Terrorism and Homeland Security	https://sites.duke.edu/tcths/#
Department of Homeland Security	https://www.dhs.gov/topics
Public Safety Canada	https://www.publicsafety.gc.ca/index-en.aspx
National Consortium for the Study of Terrorism and Responses to Terrorism (START)	https://www.start.umd.edu/
Terrorism Research Centre	http://www.terrorism.org/
Global Centre on Cooperative Security	https://www.globalcenter.org/publications/
Hedayah	http://www.hedayahcenter.org/publications
RAND Corporation	https://www.rand.org/topics/terrorism.html?content-type=research
Radicalisation Awareness Network (RAN)	https://ec.europa.eu/home-affairs/what-we-do/networks/radicalisation_awareness_network_en
RadicalisationResearch	https://www.radicalisationresearch.org/
Royal United Services Institute (RUSI)	https://rusi.org/
Impact Europe	http://impacteurope.eu/
National Criminal Justice Reference Service	https://www.ncjrs.gov/library.html
Terrorism Research Centre (University of Arkansas)	https://terrorismresearch.uark.edu
International Association of Law Enforcement Intelligence Analysts	https://www.ialeia.org
Naval Post‐Graduate School	https://nps.edu

### 3.3 Description of methods used in primary research

Existing literature highlights a growing range of police multiagency approaches that address terrorism, radicalisation, and/or extremism (e.g., see Butt & Tuck, 2014; Wise, Roberts, Formosa, & Chan, 2018). We anticipate that studies captured under the review of intervention effectiveness (Objective 1) will utilise quasiexperimental research designs (e.g., Williams, Horgan, & Evans, 2016). We anticipate that studies captured under the review of mechanisms, moderators, implementation and economic considerations (Objective 2) will utilise quantitative and qualitative data collected and presented in a range of formats, such as analysis of implementation documentation, interviews or focus groups, case studies or cost analyses (e.g., Butt & Tuck, 2014; Mabrey, Hepner, & Ward, 2006; Mastroe, 2016).

### 3.4 Assessment of risk of bias in included studies

#### 3.4.1 Assessment of risk of bias in included studies for review of effectiveness (Objective 1)

For studies included in the synthesis of intervention effectiveness (Objective 1), risk of bias will be evaluated using either the Cochrane randomised or nonrandomised risk of bias tools, depending on the study methodology (Higgins et al., 2019). Using these tools, studies will be rated across domains as having high, low or unclear risk of bias. Study authors will be approached to obtain missing data where a domain is rated as “unclear”. Results of the risk of bias assessment will be presented in summary tables and in a risk of bias summary figure. Depending on available data, sensitivity analysis will be used to examine the impact of risk of bias on effect estimates and corresponding confidence intervals. Possible analyses will include forest plots stratified by level of risk, moderator analysis or meta‐regression. The level of variation in risk of bias across included studies will determine the approach to incorporate risk of bias into statistical analyses. For example, statistical analysis may be stratified by level of risk or all studies may be included in one analysis with a narrative discussion of the risk of bias (see Higgins et al., 2011 for more detail).

#### 3.4.2 Assessment of risk of bias in included studies for review of mechanisms, moderators, implementation and economic considerations (Objective 2)

The methods and approach for critically appraising studies included in qualitative syntheses is a contentious issue in the literature (see Dixon‐Woods et al., 2006; Dixon‐Woods, Agarwal, Jones, Young, & Sutton, 2005; Noyes et al., 2019; Spencer et al., 2004). Although the studies included in the review of mechanisms, moderators, implementation and economic considerations (Objective 2) will not be used to inform estimates of effectiveness, it will be important to assess the quality of the research that will underpin conclusions drawn from the synthesis. Therefore, we will extract and summarise information on the research design and general methodological approach of each study included in the review of mechanisms, moderators, implementation and economic considerations (Objective 2). In addition, we will appraise each study deemed eligible for the review of mechanisms, moderators, implementation and economic considerations (Objective 2) using a modified version of the CASP qualitative appraisal tool (Critical Appraisal Skills Programme, 2018; see Supporting Information Appendix C). Studies will be rated as low‐quality and not included in the synthesis if the answer to the following items on the CASP tool are “No” or “Can't tell”: (a) Is the research design appropriate to answer the question?; and (b) Was the sampling strategy appropriate to the aims of the research? (see Higginson et al., 2015). While affirmative responses to these questions do not guarantee a study is of high quality, taking this approach will ensure that the research syntheses under Objective 2 will have some level of reliability. Finally, if the review identifies cost‐benefit analyses, we will assess the quality of these studies using Evers et al. (2005) tool as per the recommendation of the Campbell Collaboration Economic Methods Policy Brief (Shemilt et al., 2008).

### 3.5 Criteria for determination of independent findings

We will use a common process for the review of effectiveness (Objective 1) and the review of mechanisms, moderators, implementation and economic considerations (Objective 2). Issues of dependence can arise where: (a) multiple documents report on a single empirical study, (b) multiple conceptually similar outcomes are reported in the one document and/or (c) studies have clustering in their research design. For meta‐analyses, each eligible study will be included only once for each conceptually distinct outcome category.

The software that will be used for this review (*SysReview*; Higginson & Neville, 2014) allows the nesting of multiple dependent documents relating to a single study, to ensure that multiple reports of the same study are identified and coded appropriately. We will be alerted to possibly dependent studies where there are similar authors and highly similar descriptions of the intervention, participants, funding and implementation considerations (e.g., time period) and references to other reports of the study. If we identify dependent documents that report on the one study, we will code all documents and the most complete report of the study will be used for data extraction. If necessary and where appropriate, data may be extracted from multiple documents to enable the calculation of effect sizes.

If documents report on multiple conceptually similar outcomes, we will average these effect sizes and the averaged effect size will be included in the meta‐analysis using the approach suggested by Borenstein et al. (2009). If studies use a clustered research design (e.g., study sites assigned to conditions), the method proposed by Fu et al. (2013) and Higgins et al. (2011) will be used to adjust the standard error of the effect sizes (SE). If document authors do not report the required intra‐class correlation coefficient (ICC), we will conduct sensitivity analyses to explore whether the results of the meta‐analyses change with ICCs of 0, 0.03, 0.02, and 0.1 (Barlow, Bergman, Kornør, Wei, & Bennett, 2016).

### 3.6 Selection of studies

#### 3.6.1 Overview

We will use a common screening process to assess study eligibility for the review of effectiveness (Objective 1) and the review of mechanisms, moderators, implementation and economic considerations (Objective 2). We will first conduct initial title and abstract screening, followed by full‐text eligibility screening. At the full‐text eligibility screening stage we will categorise each study by its eligibility for inclusion in either (a) the review of effectiveness; (b) the review of mechanisms, moderators, implementation and economic considerations; or (c) both objectives. Full details of the study selection process are below.

#### 3.6.2 Title and abstract screening

The first stage of assessing study eligibility will begin with screening the titles and abstracts of all unique records captured by the systematic search. After removing duplicates and ineligible document types (e.g., book reviews, blog posts) from the results of the systematic search, all records will be imported into the review management software, *SysReview* (Higginson & Neville, 2014). Each title and abstract (record) will then be assessed according to the following exclusion criteria:

1. Ineligible document type
2. Document is not unique
3. Document is not about policing radicalisation, terrorism or extremism

Although all efforts will be made to remove ineligible document types and duplicates prior to screening, we acknowledge that automated and manual cleaning may miss some duplicates or ineligible document types. Therefore, the first two exclusion criteria will be used to remove ineligible document types and duplicates prior to screening each record on substantive content relevance. It is important to note that “policing” is broadly operationalised in both the GPD screening and the screening for this review. Specifically, a title and abstract can be screened as being about policing if, for example: police are study participants, police are involved in implementing an intervention (alone or in partnership with others), of the focus of the research appears to be police tools, technologies or techniques (see Higginson et al., 2015).

Two review authors (E. E. and L. H.) will train research staff to screen the titles and abstracts using a standardised screening companion. Each review author or research staff member conducting title and abstract screening will be required to screen the same set of 50 records and their answers will be compared against the answers determined by review authors (E. E. and L. H.). Feedback will be provided to all screeners based on their results prior to them beginning independent screening. A random sample of 5% of each screener's exclusion screenings will be cross‐checked to identify high rates of false negative screening decisions. If a screener's decisions are deemed unreliable due to a high rate of false negatives, their exclusion screenings will be reassigned to another screener.

The majority of records indexed in the GPD have a pre‐existing full‐text document and will not require retrieval. Records from the supplementary searches that are deemed as potentially eligible at the title and abstract screening stage will progress to a literature retrieval stage, where we will attempt to locate the full‐text document. If full‐text documents cannot be located via existing university resources, we will order the document through the university libraries of the review authors or by contacting study authors. All potentially eligible records will then progress to full‐text eligibility screening.

#### 3.6.3 Full‐text eligibility screening

We will screen the full‐text of each document for final eligibility using a two‐stage process. The following exclusion criteria will be used for the first stage of screening:

1. Ineligible document type
2. Document is not unique
3. Document does not include an empirical study of a multiagency intervention with police as a partner that aims to address radicalisation, terrorism or extremism

While all efforts will be made to remove ineligible document types and duplicate documents in earlier stages, these types of records can occasionally progress into later stages of screening (e.g., where duplicate records are not adjacent to each other during screening or where screeners cannot unequivocally determine the document type based on the title and abstract). Therefore, the first two exclusion criteria will be used to remove ineligible document types and duplicates.

The purpose of the second stage of screening will be to categorise studies according to the review objectives. Specifically, screeners will be asked to determine whether each study is (a) a quantitative impact evaluation of an eligible intervention, using an eligible research design, eligible outcomes, and eligible participants; (b) a qualitative study describing the implementation of an eligible intervention or (c) a study that is both quantitative and qualitative.

Two review authors (E. E. and L. H.) will train research staff to screen the documents using a standardised screening companion. Each review author or research staff member conducting full‐text document screening will be required to screen the same set of 25 documents and their answers will be compared against the answers determined by two review authors (E. E. and L. H.). Feedback will be provided to all screeners based on their results prior to them beginning independent screening. A random sample of 5% of each screener's exclusion screenings will be cross‐checked to identify high rates of false negative screening decisions. If a screener's decisions are deemed unreliable due to a high rate of false negatives (≥5%), their exclusion screenings will be reassigned to another screener. Any disagreements in determining a study's final eligibility for the review will be resolved via discussion with a third review author (A. H.).

### 3.7 Data extraction and management

Eligible documents progressing from the full‐text screening stages will be coded within *SysReview*, using the coding companion provided in Supporting Information Appendix C. The level of coding will depend on the category to which it is assigned. Data pertaining to the general study characteristics (e.g., document type, study location) will be extracted for studies included in the review of effectiveness (Objective 1) and the review of mechanisms, moderators, implementation and economic considerations (Objective 2).

For studies eligible for the review of effectiveness (Objective 1), data will be extracted according to the following general domains:

1. Participants (e.g., sample characteristics by condition, attrition)
2. Intervention (e.g., intervention components, intensity, setting)
3. Outcomes (e.g., conceptualisation, mode of measurement, time‐points)
4. Research methodology (e.g., design, unit and type of assignment)
5. Effect size data
6. Risk of bias

For studies eligible for the review of mechanisms, moderators, implementation and economic considerations (Objective 2), data will be extracted according to the following general domains (see also “Treatment of qualitative research” section):

1. Research approach (e.g., design, sampling)
2. Participant characteristics
3. Mechanisms that may explain intervention outcomes
4. Moderators that may impact intervention outcomes
5. Implementation considerations (e.g., barriers or facilitators)
6. Economic considerations

We anticipate that some studies included in the quantitative component of the review may report information to the qualitative component of the review, yet this information may not be collected, analysed or reported in the same way as the effectiveness data. Therefore, studies eligible for both the quantitative and qualitative components of the review, data will be extracted according to both of the abovementioned frameworks.

Two review authors (E. E. and L. H.) will train research staff to code the documents using a standardised coding companion. Each review author or research staff member conducting data extraction and coding will be required to code the same set of five documents and their answers will be compared against the answers determined by two review authors (E. E. and L. H.). Feedback will be provided to all coders based on their results prior to them beginning independent coding. All data extracted for effect size calculations will be independently double‐coded to minimise potential errors and/or bias. Any coding disagreements will be resolved via discussion with a third review author (A. H.).

### 3.8 Statistical procedures and conventions (Objective 1)

Studies with sufficient data to calculate effect sizes will be included in the meta‐analyses in the review of effectiveness (Objective 1). We will contact study authors to obtain missing data if their study report contains insufficient information to calculate effect sizes. If we cannot obtain the missing data, the study will be not be included in the meta‐analyses, but will still be included in the narrative description of eligible studies. Meta‐analyses will be performed for all outcomes where there are at least two independent effect sizes, however, we will conduct separate meta‐analyses: (a) for each set of conceptually similar outcomes; and (b) where participants are individuals and studies where participants are places, even if both groups of studies report on the same outcome. We will use either *Revman*, or *Stata* for more complex data structures (StataCorp, 2019) to conduct random effects inverse variance meta‐analyses (Lipsey & Wilson, 2001) and will report mean effect sizes and their corresponding confidence intervals in‐text and graphical forest plots.

We will follow Campbell Collaboration guidelines and plan to transform the smallest number of effect sizes to a common effect size (Polanin & Snilstveit, 2016), so that the final effect size metric will be that which is calculated most commonly for each outcome. Where the participants are individuals, we anticipate that evaluations will typically report outcomes as continuous measures (e.g., willingness to engage in violence, self‐reported on a Likert scale), and in these instances, Hedges' *g* (standardised mean differences, SMDs) will be computed. Where evaluations report binary outcomes (e.g., disengagement from radicalised peers: yes/no), effect sizes will be computed as odds ratios, and then transformed into Hedges' *g* for meta‐analyses (see Borenstein et al., 2009).

Where the participants are micro‐ or macro‐places, we anticipate that evaluations may report outcomes as counts or rates in the intervention and comparison areas, before and after the intervention (e.g., number of radicalised individuals). In these instances, we will calculate the effect size as the relative incident rate ratio (RIRR), which can be interpreted as the relative proportion change in the outcome in the treatment area after the intervention, compared to the comparison area (Farrington, Gill, Waples, & Argomaniz, 2007; Higginson & Mazerolle, 2014). RIRR is calculated as:

RIRR=ad/bc,

where *a* = count or rate in the intervention area before the intervention, *b* = count or rate in the intervention area after the intervention, *c* = count or rate in comparison area before the intervention and *d* = count or rate in the comparison area after the intervention. The RIRR will be converted to a log relative incident rate ratio (LRIRR) for synthesis, but converted back to RIRR for more intuitive reporting. The standard error of the LRIRR is initially calculated as:

$${\mathrm{se}}_{\log(\text{RIRR})}=\sqrt{\frac{1}{a}+\frac{1}{b}+\frac{1}{c}+\frac{1}{d}},$$

however, the odds ratio formula of the RIRR assumes a Poisson distribution, to which crime data typically does not conform. Therefore, following Wilson (forthcoming) we will calculate an overdispersion parameter (ϕ) which is a function of the means and standard deviations of the counts, assuming a quasi‐Poisson distribution:

$$\emptyset =\left(\frac{1}{\sum n_{k}-4}\right)\sum \frac{s_{k}^{2}\left(n_{k}-1\right)}{{\bar{x}}_{k}},$$

where $n_{k}$ is the number of counts in each of *a* to *d*, ${\overset{®}{x}}_{k}$ is the mean of each count and $s_{k}$ is the standard deviation for each of the mean counts (Wilson, forthcoming). Where ∅≤1 there is no overdispersion under the Poisson distribution. If ∅>1, there is overdispersion, so we will adjust the standard error by first multiplying ${\mathrm{se}}^{2}$ by ∅ and then taking the square root (Wilson, forthcoming):

$$\begin{array}{c} {\mathrm{se}}_{\text{adj}}\;=\; \end{array}\left\{ \begin{array}{ccc} \sqrt{{\mathrm{se}}^{2}\times \emptyset } & if & \emptyset >1,\\ \mathrm{se} & \text{if} & \emptyset \leq 1. \end{array}\right.$$

We will conduct separate meta‐analyses for each of the reported follow‐up time points, where data permits. Where studies report multiple points of follow‐up, effect sizes will be calculated for each time‐point, but synthesised separately with studies that have similar outcome time‐points. If studies report both baseline and postintervention outcome data, SMDs will be calculated using baseline adjusted mean differences (i.e., mean change scores) and the change score standard deviations will be standardised using the raw standard deviation within groups. If the standard deviation for mean change scores is not available, we will follow Lipsey and Wilson's (2001) formula to calculate the standard deviation. If studies report follow‐up outcome data, post‐only outcome data will be used to estimate SMDs, and follow‐up outcomes will be analysed separately from postintervention outcomes.

For each meta‐analysis, we will assess heterogeneity in effect sizes using the *I* ^2^ statistic, *χ* ^2^ test, and *τ* ^2^ (Higgins & Thompson, 2002). We will conduct moderator analyses to explore potential sources of heterogeneity, using the variables listed under Objectives. The analogue to analysis of variance will be used for categorical moderators and regression‐based approaches will be used for continuous moderators. We may also conduct additional exploratory subgroup analyses, but will clearly distinguish between a priori and post hoc analyses in our reporting.

Finally, we will visually and statistically assess the data for evidence of publication bias. We will inspect funnel plots for asymmetry, and if asymmetry is detected, we will conduct subgroup analyses to assess if the effect sizes from the published and unpublished documents are significantly different.

### 3.9 Treatment of qualitative research (Objective 2)

For the review of mechanisms, moderators, implementation and economic considerations (Objective 2) we will draw on the realist synthesis informed EMMIE framework developed by the United Kingdom's *What Works for Crime Reduction Centre* (Johnson, Tilley, & Bowers, 2015; Thornton, Sidebottom, Belur, Tompson, & Bowers, 2019). This framework aims to structure the extraction and discussion of the **E**ffects of an intervention, the **M**echanisms by which the intervention is believed to work, the **M**oderators that may vary intervention effectiveness (e.g., characteristics of target people or places), **I**mplementation considerations (e.g., required resources, training) and **E**conomic implications for the intervention in terms of costs and benefits (Johnson et al., 2015; Thornton et al., 2019). The review of effectiveness (Objective 1) encompasses the Effectiveness part of the EMMIE framework, so the qualitative component of this review (Objective 2) will focus on qualitatively synthesising the mechanisms, moderators, implementation and economic domains. The data extraction for these domains (Supporting Information Appendix C) will be adapted from the EMMIE codebook (Tompson, Bowers, Johnson, & Belur, 2015) which has been utilised in number of realist‐informed systematic reviews (e.g., Belur et al., 2017; Sidebottom et al., 2015; see also Gielen, 2015) and for rating the evidence of systematic reviews in the area of criminal justice (https://whatworks.college.police.uk/toolkit/Pages/Welcome.aspx).

There are multiple approaches available for qualitative synthesis, yet the development of a clear set of guidelines has been a complex and long‐term problem (Booth et al., 2018; Noyes et al., 2019) and many of the methods have not been thoroughly evaluated for use in mixed‐methods systematic reviews (Dixon‐Woods et al., 2005, 2006; Popay et al., 2006; Pope, Mays, & Popay, 2007). Explicitly labelling our planned qualitative synthesis approach is also complicated by the variations in the terminology in the literature and significant overlap in techniques within different synthesis approaches (Booth et al., 2016; Pope et al., 2007).

We will use a Framework Synthesis approach to synthesise the qualitative data (see Booth et al., 2016), which is an overarching approach that encompasses analogous methods such as content analysis, framework analysis, and aggregate synthesis (see Booth & Carroll, 2015; Booth et al., 2016; Dixon‐Woods et al., 2005, 2006; Dixon‐Woods, 2011; Noyes et al., 2019; Popay et al., 2006). Broadly, these methods use systematic rules or a framework to arrange data into distinct categories that are then synthesised using a variety of techniques such as tables, matrices, and narrative textual summaries (e.g., see: Belur et al., 2017; Petrosino, Morgan, Fronius, Tanner‐Smith, & Boruch, 2012; Sidebottom et al., 2015). For the purposes of our review, we will extract data from the studies eligible for the qualitative component of the review using the coding framework provided in Supporting Information Appendix C according to the following categories: general study details, research approach, participants, mechanisms, moderators, implementation considerations, and economic information. The extracted qualitative data will then be synthesised in distinct sections pertaining to the mechanism, moderator, implementation and economic domains. Specifically, within each domain subsection, narrative text and tables will be used to summarise the number of studies reporting data for that domain, the types research approaches used by included studies (e.g., design and participants), and overarching themes across the corpus of studies within the relevant domain. For example, the synthesis of implementation considerations may contain subsections summarising empirical research findings about the barriers and facilitators that arise during the implementation of police‐involved multiagency interventions for countering terrorism, violent extremism or radicalisation to violence.

## ROLES AND RESPONSIBILITIES

- Content: Mazerolle, A. C., E. B., E. E., and L. H.
- Systematic review methods: E. E., A. H., and L. H.
- Statistical analysis: A. H. and E. E.
- Information retrieval: E. E., A. H. and L. H.

## FUNDING

This review is funded by a Campbell Collaboration grant awarded to Lorraine Mazerolle, Elizabeth Eggins, Adrian Cherney, and Angela Higginson via Public Safety Canada. The final review is due for submission to the Campbell Collaboration in 2020.

## POTENTIAL CONFLICTS OF INTEREST

Three of the review authors have internal roles within the Campbell Collaboration Crime and Justice Group. Lorraine Mazerolle is the Co‐Chair of the Crime and Justice Coordinating Group (CJCG), Angela Higginson is the Editor of the CJCG, and Elizabeth Eggins is the Managing Editor of the CJCG. Consequently, Mazerolle, Higginson and Eggins will not be involved in any editorial or internal Campbell Collaboration communications about this review. In addition, Adrian Cherney has published research that is closely linked with the review topic. To minimise potential bias, Cherney will not be involved in the screening or coding of any studies for this review.

## PRELIMINARY TIMEFRAME

We plan to submit the final review by July 2020.

## PLANS FOR UPDATING THE REVIEW

Lorraine Mazerolle and Adrian Cherney will be responsible for updates of this review, which are anticipated to occur every 3–5 years.

## Footnotes

1 We conducted a search of the literature using the following terms to identify existing reviews: terroris* OR extremis* OR radicali*. Searches of the following locations did not identify any existing systematic reviews (completed or ongoing) on the specific topic proposed in this proposal: Campbell Collaboration; Cochrane Collaboration; PROSPERO registry; Google Scholar.
